# Aminoglycoside use in paediatric febrile neutropenia – Outcomes from a nationwide prospective cohort study

**DOI:** 10.1371/journal.pone.0238787

**Published:** 2020-09-16

**Authors:** Brendan J. McMullan, Gabrielle M. Haeusler, Lisa Hall, Louise Cooley, Andrew J. Stewardson, Christopher C. Blyth, Cheryl A. Jones, Pamela Konecny, Franz E. Babl, Françoise Mechinaud, Karin Thursky, on behalf of the Australian PICNICC study group and the PREDICT network

**Affiliations:** 1 NHMRC National Centre for Infections in Cancer, University of Melbourne, Melbourne, Victoria, Australia; 2 Department of Immunology and Infectious Diseases, Sydney Children’s Hospital, Randwick, Sydney, NSW, Australia; 3 School of Women’s and Children’s Health, University of New South Wales, Sydney, NSW, Australia; 4 Department of Infectious Diseases, Peter MacCallum Cancer Centre, Melbourne, Victoria, Australia; 5 Sir Peter MacCallum Department of Oncology, University of Melbourne, Parkville, Victoria, Australia; 6 The Paediatric Integrated Cancer Service, Parkville, Victoria, Australia; 7 Infection Diseases Unit, Department of General Medicine, Royal Children’s Hospital, Parkville, Victoria, Australia; 8 Infection and Immunity Theme, The Murdoch Children’s Research Institute, Parkville, Victoria, Australia; 9 School of Public Health, University of Queensland, Brisbane, Queensland, Australia; 10 Department of Microbiology and Infectious Diseases, Department of Medicine, University of Tasmania, Hobart, Tasmania, Australia; 11 Department of Infectious Diseases, The Alfred and Central Clinical School, Monash University, Melbourne, Victoria, Australia; 12 School of Medicine, University of Western Australia, Perth, Western Australia, Australia; 13 Wesfarmers Centre of Vaccines and Infectious Diseases, Telethon Kids Institute, Perth, Western Australia, Australia; 14 Department of Paediatric Infectious Diseases, Perth Children's Hospital, Perth, Western Australia, Australia; 15 Department of Microbiology, PathWest Laboratory Medicine, QEII Medical Centre, Perth, Western Australia, Australia; 16 Sydney Medical School, Faculty of Medicine and Health, University of Sydney, Sydney, NSW, Australia; 17 Sydney Children’s Hospital Network–The Children’s at Westmead, Westmead, NSW, Australia; 18 Department of Paediatrics, University of Melbourne, Melbourne, Victoria, Australia; 19 Murdoch Children’s Research Institute, Parkville, Victoria, Australia; 20 Department of Infectious Diseases, Immunology & Sexual Health, St George Hospital, Sydney, NSW, Australia; 21 St George & Sutherland Clinical School, University of New South Wales, Sydney, NSW, Australia; 22 Department of Clinical Sciences, Murdoch Children's Research Institute, Parkville, Victoria, Australia; 23 Emergency Department, Royal Children's Hospital, Parkville, Victoria, Australia; 24 Paediatric Research in Emergency Departments International Collaborative (PREDICT), Parkville, Victoria, Australia; 25 Royal Children's Hospital, Parkville, Victoria, Australia; 26 Hôpital Robert Debré APHP Nord-Université de Paris, Paris, France; 27 Department of Medicine, University of Melbourne, Parkville, Victoria, Australia; 28 NHMRC National Centre for Antimicrobial Stewardship, The Peter Doherty Institute for Infection and Immunity, Melbourne, Victoria, Australia; Rabin Medical Center, Beilinson Hospital, ISRAEL

## Abstract

Aminoglycosides are commonly prescribed to children with febrile neutropenia (FN) but their impact on clinical outcomes is uncertain and extent of guideline compliance is unknown. We aimed to review aminoglycoside prescription and additional antibiotic prescribing, guideline compliance and outcomes for children with FN. We analysed data from the Australian Predicting Infectious ComplicatioNs in Children with Cancer (PICNICC) prospective multicentre cohort study, in children <18 years with FN between November 2016 and January 2018. Impact of aminoglycoside use in the first 12 hours of FN on composite unfavourable outcome of death, ICU admission, relapse of infection or late-onset sepsis was assessed using multivariable Cox regression. The study was conducted in Australia where antimicrobial resistance among gram negative organisms is relatively low. Data from 858 episodes of FN in 462 children from 8 centres were assessed, median age 5.8 years (IQR 3.5–10.8 years). Early empiric aminoglycosides were prescribed in 255 episodes (29.7%). Guideline non-compliance was common: in 46% (184/400) of eligible episodes, patients did not receive aminoglycosides, while aminoglycosides were prescribed in 9% (39/458) of guideline-ineligible episodes. Adjusted hazard of the composite unfavourable outcome was 3.81 times higher among patients prescribed empiric aminoglycosides than among those who weren’t (95% confidence interval, 1.89–7.67), with no increased risk of unfavourable outcome in eligible patients who did not receive aminoglycosides. In a large paediatric FN cohort, aminoglycoside prescription was common and was often non-compliant with guidelines. There was no evidence for improved outcome with aminoglycosides, even in those who met guideline criteria, within a low-resistance setting. Empiric aminoglycoside prescription for children with FN requires urgent review in guidelines and in national practice.

## Introduction

Antibiotics are prescribed for children with febrile neutropenia (FN) undergoing treatment for malignancy to reduce morbidity and mortality. More than 80% of children with acute leukaemia and more than 50% of children with a solid tumour develop FN at least once during the course of their treatment [1]. While fever may signal a life-threatening infection, invasive bacterial infections are identified in only a minority of patients with FN [2, 3].

While data from surveys conducted across the United Kingdom [4], Australia [5] and North America [6] indicate variation in approaches to empiric aminoglycoside prescribing, little is known about real world effects of this variation on patient outcomes [5]. Aminoglycosides are commonly given to patients who are clinically unstable or where there is a concern about antimicrobial resistance [7]. Routine addition of an aminoglycoside, however, may be associated with additional costs for patients and healthcare facilities and potentially negatively affect outcomes due to medication side-effects [8]. Recent international paediatric FN guidelines recommend monotherapy with an antipseudomonal β-lactam or cephalosporin for empiric treatment of high-risk FN [7]. Based on data from randomized controlled trials of FN in children, which failed to demonstrate a benefit (or harm) with empiric aminoglycoside therapy added to a β-lactam, guidelines recommend reserving addition of a second gram-negative agent for “patients who are clinically unstable, when a resistant infection is suspected, or for centres with a high rate of resistant pathogens” [9]. Implementation of and adherence to evidence-based guidelines might reduce unnecessary variation from best practice care but this has not been studied in detail in the context of pediatric FN [10].

The Australian Predicting Infectious ComplicatioNs in Children with Cancer (PICNICC) study is a prospective, multisite cohort study that enrolled children with cancer and FN from all eight tertiary paediatric hospitals throughout Australia [11]. The primary aim of the PICNICC study was to validate clinical decision rules (CDRs) that stratify children (aged ≤18 years) with a diagnosis of cancer or leukaemia (on active treatment) and FN into low- and high-risk groups for infection or adverse outcome. The PICNICC study also collected data on empiric antibiotic use, making it a valuable dataset for analysis of prescribing variation, guideline compliance and outcomes in this patient group.

The objectives of this analysis are to (1) compare initial empiric aminoglycoside and other antibiotic prescribing in relation to local guidelines, and (2) determine impact of aminoglycoside prescribing on patient outcomes.

## Materials and methods

The Australian PICNICC study is a prospective cohort study, in which children (aged ≤18 years) with a diagnosis of cancer or leukaemia on active treatment and FN were enrolled consecutively [11]. The study was open to recruitment between November 2016 and January 2018. Recruitment took place at 8 Australian tertiary paediatric hospitals which have cancer centres, specifically within the emergency departments and wards of these hospitals. Patients were consecutively enrolled by study doctors and research nurses and multiple episodes of FN per patient were allowed. Episodes were excluded if FN treatment commenced at a non-participating site, if the patient had undergone an allogeneic hematopoietic stem cell transplant (HSCT) within the preceding three months or if the episode occurred while in receipt of concurrent intravenous or oral antibiotics (excluding antibiotic prophylaxis). The unit of analysis presented here is FN episode.

Detailed demographic, FN episode and outcome data were collected prospectively by study-site research assistants (RAs), following international consensus definitions.(12) Information on up to 4 antibiotics prescribed in the first 12 hours of each FN episode was collected, with antibiotics listed in the order they were administered. For the first antibiotic administered, dose, frequency, route and start time were also collected. For each episode, we assessed compliance with locally applicable guidelines for use of aminoglycosides Table 1. We determined whether episodes met guideline criteria for aminoglycosides and also whether an aminoglycoside was actually prescribed in the first 12 hours of FN episode.

**Table 1:** Antibiotic recommendations for paediatric febrile neutropenia by site.

State^a^	First-line β-lactam^b^	Aminoglycoside indications^c^
NSW (3 sites)	Piperacillin-tazobactam	Gentamicin routinely recommended
QLD (1 site)	Piperacillin-tazobactam	Add gentamicin if critically ill^d^
SA (1 site)	Piperacillin-tazobactam	Add gentamicin if hypotension and/or shock is present^e^
VIC (2 sites)	Piperacillin-tazobactam	Add amikacin if inpatient onset FN, systemic compromise or high-risk protocol^f^
WA (1 site)	Piperacillin-tazobactam	Add gentamicin if there is systemic compromise^g^

^a^Abbreviations are as follows: NSW = New South Wales; QLD = Queensland; SA = South Australia; VIC = Victoria; WA = Western Australia.

^b^For patients not allergic to β-lactams. During the study period there was a global shortage of piperacillin-tazobactam with resultant modifications in empiric antibiotic recommendations for some sites: the 3 NSW sites changed recommended first-line β-lactam to cefepime and 1 of 2 sites from Victoria changed to ceftazidime and flucloxacillin. Recommendations for aminoglycosides remained unchanged at all sites.

^c^Guidelines were sourced from EviQ (www.eviq.org.au/) except for SA–SA guideline provided by Dr Celia Cooper, Women’s and Children’s Hospital, SA.

^d^QLD: “critically ill” includes any of: need for fluid bolus/inotrope (including bolus >/ = 10ml/kg), or respiratory support (any), or altered conscious state

^e^SA: hypotension, as per VicTOR https://www.bettersafercare.vic.gov.au/reports-and-publications/victorian-childrens-tool-for-observation-and-response-victor Shock, as per Goldstein et al. [12]

^f^High risk protocols: Acute myeloid leukaemia (AML) treatment; Acute lymphoblastic leukaemia (ALL) induction, ALL delayed intensification, infant ALL; lymphoma induction; allogeneic transplant (day-14 to day+356); autologous transplant (day-7 to day +30); re-induction chemotherapy for any relapse. systemic compromise: includes any of need for fluid bolus/inotrope (bolus >/ = 10ml/kg), or respiratory support (any), or altered conscious state.

^g^”Systemic Compromise” includes: Haemodynamic compromise (severe sepsis/septic shock, inotrope or bolus >/ = 10ml/kg), Respiratory compromise (any resp support); Confusion or decreased consciousness; End organ dysfunction–renal or hepatic dysfunction, coagulopathy.

Aminoglycoside administration was based on hospital guidelines: dosing was according to hospital-based FN guidelines and ranged from 6–7.5 mg/kg/dose 24 hourly for gentamicin, depending on age and hospital, and 18–22.5 mg/kg/dose 24-hourly for amikacin, depending on age. Since our data include aminoglycoside prescribing in the first 12 hours of FN episode only, this is taken to represent empiric aminoglycoside usage, and is reviewed in reference to the hospital guidelines shown in Table 1. Aminoglycoside data are presented for the beginning of each FN episode and only once during each discrete episode. A FN episode was considered to be resolved when patient was afebrile for >48 hours and neutropenia recovered to absolute neutrophil count (ANC) of >500 cells/mm^3^. All sites recommended 48h maximum empiric use of aminoglycosides, with treatment of confirmed infection at the discretion of the treating physician. Bloodstream infections associated with these FN episodes are reported in detail elsewhere [13].

The primary composite outcome was time to death, relapse of clinical or microbiologically defined infection, admission to intensive care unit, or late-onset severe sepsis (with onset greater than 4 hours post initial FN presentation or onset). Patients were followed-up for 30-days from FN onset.

### Definitions

To reflect standard practice across all eight participating institutions, we included patients with fever >38.0 and ANC<1000 cells/mm^3^. Severe sepsis was defined according to Goldstein *et al*. [12] ‘Unwell’ appearance was defined as severe sepsis or septic shock [12], altered conscious state (Glasgow Coma Score <15 or only responsive to voice or pain), documentation the patient was ‘severely unwell’ or equivalent in patient record, or either blood pressure or respiratory rate within the mandatory emergency call range [11]. Episodes were also stratified into low- and high-risk using the Swiss Paediatric Oncology Group (SPOG) rule [14, 15]. The SPOG rule takes into account chemotherapy intensity, haemoglobin, white cell count and platelet count and the score predicts serious medical complication as a result of infection, microbiologically-defined infection or radiologically-confirmed pneumonia in children with FN.

### Statistical methods

Categorical variables including antibiotic choice and guideline compliance are reported as frequencies and percentages. Median and interquartile ranges (IQRs) are reported for continuous variables. Univariate analysis of categorical variables was conducted using χ2 and Fisher exact tests. The threshold for significance was taken as 0.05. Time to first instance of composite unfavourable outcome is calculated in whole days, except where these events occurred on the first day of enrolment: these were calculated in decimal fractions of 1 day (24 hours). We used multivariable Cox proportional hazards regression to quantify the impact of empiric aminoglycoside use on the primary composite outcome. The following potential confounders judged most clinically relevant were included in the model: risk status using the SPOG rule, severe sepsis at presentation, age, hypotension at presentation, ‘unwell’ status at presentation, receipt of fluid bolus (>10ml/kg), study site and time to first antibiotic. For survival analysis of hospital length of stay, Wilcoxon rank sum test was used. Propensity matching was performed as a secondary analysis with 1:1 matching, to provide an additional estimate of the effect of aminoglycoside prescription on composite unfavourable outcome, with the same baseline variables as for the Cox regression. We performed additional sensitivity analyses adjusting for malignancy type and first vs. subsequent episode of FN per patient in the dataset. Two further sensitivity analyses included adjusting for patients with any bloodstream infections and those with gram-negative bloodstream infections in Cox regression. Statistical analysis was performed using Stata, version 16.0 (StataCorp LP).

### Ethics statement

The PICNICC study had national Human Research Ethics Committee approval, No. 36040A from The Royal Children’s Hospital Human Research Ethics Committee. Written informed consent was obtained from parents or guardians, with assent obtained where possible from children/adolescents depending on maturity and capacity to understand the study. A patient information and consent form was signed by a parent/guardian and witnessed by a study investigator at each site.

## Results

There were 858 FN episodes among 462 patients enrolled from eight sites in five states within Australia: 427 episodes (49.8%) from Victoria (VIC), 173 (20.2%) from Queensland (QLD), 162 (18.9%) from New South Wales (NSW), 67 (7.8%) from South Australia (SA) and 29 (3.4%) from Western Australia (WA). Overall 51.6% (443) were male and the median age was 5.8 years (IQR 3.5–10.8 years). As described above, we included all episodes of ANC<1000/mm^3^ but the majority had ANC <500/mm^3^. Only 85 episodes (10%) had initial ANC 500-<1000 and 64 of these (75%) decreased within 24 hours.

Demographic and clinical criteria for patients are summarised in Table 2.

**Table 2:** Demographic and clinical criteria for febrile neutropenia episodes by receipt of aminoglycoside.

Variable	Received early aminoglycosides (n = 255)	Did not receive early aminoglycosides (n = 603)	All (n = 858)
Median age, years (IQR)	6.8 (3.5–11.1)	5.7 (3.4–10.7)	5.8 (3.5–10.8)
Female, n (%)	124 (48.6)	291 (48.3)	415 (48.4)
Acute leukaemia, n (%)	163 (63.9)	286 (47.4)	449 (52.3)
Lymphoma, n (%)	21 (8.2)	45 (7.5)	66 (7.7)
Solid tumour, n (%)	71 (27.8)	272 (45.1)	343 (40.0)
Bacteraemia	60 (10.0)	51 (20.0)	111 (12.9)
High SPOG^a^ score	156 (61.2)	319 (52.9)	475 (55.4)
Early-onset sepsis (within 4 hours)	124 (48.6)	262 (43.5)	386 (45.0)
Baseline hypotension	29 (4.8)	20 (7.8)	49 (5.7)
Unwell appearance^b^	32 (12.6)	39 (6.5)	71 (8.3)
Received fluid bolus (≥10ml/kg)	52 (20.4)	47 (7.8)	99 (11.5)
Time to 1st antibiotic in minutes (median, IQR)	45 (24–63)	52 (36–75)	50 (33–71)

^a^Abbreviations are as follows: SPOG, Swiss Paediatric Oncology Group (SPOG) rule.

^b^Unwell appearance was defined as severe sepsis or septic shock, altered conscious state, documented as ‘severely unwell’ or equivalent in the patient record or either blood pressure or respiratory rate within the mandatory emergency call range.

### Aminoglycoside prescribing

A total of 255 aminoglycoside prescriptions were recorded. The most commonly prescribed aminoglycoside was amikacin (141/255 = 55.3%). Aminoglycoside prescribing varied by state: highest in NSW at 58% (94/162) of episodes and lowest in WA, where none of 29 episodes received empiric aminoglycoside therapy. This is partially explained by variation in local guideline recommendations for empiric aminoglycoside between states Table 1. Actual receipt of aminoglycoside therapy compared with local guideline-eligibility is shown in Supporting Information S1 Table. Overall 46% (n = 184) of those who met local guideline criteria based on clinical information documented did not receive an aminoglycoside. A smaller proportion who did not meet guideline criteria received an aminoglycoside: 12.2% (26/212) in VIC, 7% (11/157) in QLD, 3.2% (2/63) in SA and none in NSW or WA S1 Table.

### Empiric antibiotic prescribing

In the first 12 hours of FN episode, 855 (99.7%) received 1 antibiotic, 427 (49.8%) received 2 antibiotics, 91 (10.6%) received 3 antibiotics and 7 episodes (0.8%) received at least 4 antibiotics concurrently. Time to first antibiotic (in minutes) was available in 854/858 episodes (99.5%) and first antibiotic was given at a median of 50 minutes of FN episode onset (IQR 33–71 minutes). All antibiotics prescribed in the 1^st^ 12 hours of FN episode are listed in Table 3. Time to antibiotics for subsequent antibiotics, after the first antibiotic was given, was not available, but it is likely these empiric antibiotics were given in rapid succession, as per hospital protocols. Piperacillin-tazobactam was the most commonly prescribed antibiotic overall (519/858 episodes, 60.5%) followed by ceftazidime (209, 24.4%) and flucloxacillin (150, 17.5%).

**Table 3:** Empiric antibiotics for febrile neutropenia in the first 12 hours.

Antibiotic	Number of prescriptions^a^	% of episodes
Piperacillin-tazobactam	519	60.5%
Ceftazidime	209	24.4%
Flucloxacillin	150	17.5%
**Amikacin**	**141**	**16.4%**
Cefepime	122	14.2%
**Gentamicin**	**105**	**12.2%**
Vancomycin	83	9.7%
Cefotaxime or Ceftriaxone	15	1.8%
Meropenem	13	1.5%
**Tobramycin**	**9**	**1.1%**
Ciprofloxacin	5	0.6%
Metronidazole	3	0.4%
Teicoplanin	2	0.2%
Augmentin	1	0.1%
Azithromycin	1	0.1%
Clindamycin or Lincomycin	1	0.1%
Other	1	0.1%

^a^Patients received up to 4 concurrent antibiotics, total antibiotics received = 1380. Aminoglycosides are displayed in bold text.

### Clinical outcomes

All 858 FN episodes were followed to 30 days or until the day of death if this occurred within 30 days. In 54 episodes (6.3%) a composite unfavourable outcome of death, ICU admission, relapse of infection or late-onset sepsis occurred within 30 days. Death occurred in four FN episodes (0.5%), at 11, 13, 16 and 30 days, respectively. In 24 episodes (2.8%) the patient was admitted to ICU, there were 27 (3.1%) with relapse of infection and 6 (0.7%) with late-onset sepsis. Median time to unfavourable outcome was 10 days (IQR 2–17 days). Total analysis time at risk was 24,713 days and incidence rate of unfavourable outcome was 0.22 per 100 person-days (95% CI 0.17–0.29). Bloodstream infections associated with these FN episodes are reported in detail elsewhere [13], but there were 58/858 (6.8%) episodes of FN attributed to gram-negative bloodstream infection in total, with *E*. *coli*, *Klebsiella* species, and *Pseudomonas* species as the most common gram-negative species identified, in 19 (2.2%), 14 (1.6%) and 12 (1.4%) of episodes, respectively.

Crude and adjusted hazard ratios for composite unfavourable outcome are displayed in Table 4. Those who received an aminoglycoside were more likely to have a composite unfavourable outcome. The adjusted hazard of the composite unfavourable outcome was 3.81 times higher among patients prescribed empiric aminoglycosides than among those who were not (95% confidence interval, 1.89–7.67). When restricting the analysis only to patients for whom the local guideline recommended empiric aminoglycoside treatment, the composite unfavourable outcome was less likely to occur in patients who *did not* receive an aminoglycoside than in those who did (HR 0.26, 95% CI 0.1–0.68). Kaplan-Meier curves for a favourable outcome with and without aminoglycosides are shown in S1 Fig. Covariates were well-balanced after propensity-matching S2 Fig. In propensity score analysis, adjusting for high-risk status, sepsis at presentation, age, hypotension at presentation, “unwell” appearance at presentation, receipt of fluid bolus(≥10ml/kg), study site and time to 1^st^ antibiotic, those who received aminoglycosides were 6.9% more likely to have composite unfavourable outcome (P = 0.01). Secondary sensitivity analyses adjusting for malignancy type Table 2, first vs. subsequent episode of bacteraemia, any bloodstream infection and gram-negative bloodstream infection did not significantly change the hazard ratio for aminoglycoside in Cox regression, nor its significant association composite unfavourable outcome.

**Table 4:** Factors associated with composite unfavourable outcome using a multivariate Cox regression (n = 858 episodes).

Variable	Crude HR (95% CI)	P value	Adjusted HR (95% CI)	P value
Received Aminoglycoside	**2.47 (1.45–4.21)**^**a**^	**0.001**	**3.81 (1.89–7.67)**	**<0.001**
High SPOG^b^ score	1.64 (0.93–2.88)	0.09	1.57 (0.86–2.85)	0.138
Early-onset sepsis (within 4 hours)	1.68 (0.98–2.87)	0.06	0.98 (0.54–1.74)	0.905
Age (years)	1.02 (0.97–1.08)	0.47	0.95 (0.89–1.02)	0.132
Baseline hypotension	**4.28 (2.15–8.81)**	**<0.001**	1.48 (0.38–5.72)	0.593
Unwell appearance	**4.95 (2.73–8.98)**	**<0.001**	**2.98 (1.02–8.66)**	**0.046**
Received fluid bolus (≥10ml/kg)	**5.12 (2.95–8.9)**	**<0.001**	**3.07 (1.53–6.17)**	**0.001**
Time to 1st antibiotic (hours)	**1.45 (1.18–1.79)**	**<0.001**	**1.59 (1.30–1.96)**	**<0.001**

^a^ Bold text = statistically significant, results are stratified by study site.

^b^Abbreviations are as follows: CI, confidence interval; HR, hazard ratio. SPOG, Swiss Paediatric Oncology Group (SPOG) rule. Unwell appearance was defined as severe sepsis or septic shock, altered conscious state, documented as ‘severely unwell’ or equivalent in the patient record or either blood pressure or respiratory rate within the mandatory emergency call range.

When individual endpoints among composite outcome were assessed, those prescribed aminoglycosides were more likely to have ICU admission (17/255 vs 7/596; OR 6.08, 95% CI 2.35–17.3) whereas late-onset sepsis, relapse of infection and death were not significantly different, though numbers were small. In those prescribed an aminoglycoside, median hospital length of stay was longer: 10.6 days (IQR 5.6–19.1 days) compared with 4.7 days (IQR 2.7–9.1 days, p = 0.0001).

## Discussion

This study is the first of its kind to analyse empiric aminoglycoside use, guideline compliance and outcomes in a prospective multicentre cohort of children with FN. While caution should be used in interpreting outcomes in an observational study, we found no evidence of benefit from therapy with empiric aminoglycosides in paediatric FN, rather we found more than three times the hazard of unfavourable outcome and longer hospital stay, even after adjustment for multiple potential confounding factors.

We included patients with fever >38.0 and ANC<1000 cells/mm^3^ to reflect standard practice across all eight participating institutions. Although a lower ANC value of 500 cells/mm^3^ to define FN in children and adults has been used in some studies, a consensus definition of both fever and neutropenia in children was unable to be achieved among an international panel of paediatric clinicians, researchers and consumers [16]. In our study of 858 FN episodes, of a small minority of episodes that had ANC 500–1000 (n = 85), only 21 did not decrease within 24 hours. While these have been included in this study, we have shown previously that the restriction of analysis to patients with ANC<500 for six out of the nine rules validated did not significantly influence the results [15].

Use of aminoglycosides as combination therapy in FN in adults and children remains controversial. In a broader context, aminoglycoside breakpoints have been revised by EUCAST, with new recommendations that aminoglycosides should only be used for systemic infections at high dose and in combination with other active therapy [17]. In an era of increased detection of drug resistant organisms globally, provision of an additional agent to a beta-lactam backbone, in order to treat potential gram-negative sepsis empirically has theoretical allure. Despite this, clinical studies have failed to demonstrate a benefit with combination aminoglycoside therapy and some have even demonstrated harms [9, 18, 19]. Nor is antimicrobial resistance uniform globally: information on susceptibility and resistance in relevant gram-negative pathogens is available in Australia, the setting for our study. Although we did not collect data on antimicrobial resistance in blood cultures isolates from this study, in the most recent national report on antimicrobial use and resistance in human health, resistance among *E*. *coli* isolates was reported at ~6% for piperacillin-tazobactam and ~5% for gentamicin in 2016–2017. For *Klebsiella pneumoniae*, rates were between 7–8% and 2–3%, respectively. For *Pseudomonas aeruginosa*, resistance rates were ~6% for both agents [20]. While individual hospitals or units might have higher local resistance rates to these and other agents, overall resistance in Australia is comparatively low in global terms. This is important in interpreting our results, as aminoglycosides may have greater utility where resistance rates to the standard beta-lactam agent for febrile neutropenia are higher. Nonetheless, these results argue against liberal empiric use of aminoglycosides as combination therapy for treatment of FN in an Australian or other low-resistance setting. *E*. *coli*, *Klebsiella* species, and *Pseudomonas* species were the most common gram-negative isolates identified in this study, albeit in a small number of episodes overall. Even adjusting for those with bloodstream infections, however, did not alter our finding of harm associated with aminoglycoside use.

Recently reported evidence for lack of benefit or potential harms of aminoglycoside therapy in related populations includes that of Timotëus Deelen and colleagues, who reported on a cohort of 626 adults with gram-negative bloodstream infection in the Netherlands. They reported no improvement in 30-day mortality, despite better empiric cover of pathogens identified [21]. Rhee and colleagues report on a cohort of 17,430 adults admitted to 104 US hospitals and found higher mortality with both under- and over-treatment of bloodstream infections, according to pathogens identified in cultures [22]. In a Korean paediatric cancer cohort with FN, Lee and colleagues reported no additional benefit of amikacin addition to high-dose cefepime monotherapy [23]. Overall, there is mounting evidence calling into question the status quo of empiric aminoglycoside prescribing in children and adults with FN.

In our study, fewer patients received aminoglycosides than met local guideline criteria for this therapy. This may reflect prescriber concerns about aminoglycoside toxicity and/or lack of additional efficacy, compared with β-lactam monotherapy. Bearing in mind potential unidentified confounders in our analysis, we did not find evidence to suggest those who were eligible for aminoglycosides but did not receive them fared any worse, rather the contrary. This suggests prescribers’ clinical gestalt may have additional value in predicting outcomes. Prescribers appear to have been justified in withholding aminoglycosides for these patients despite guideline recommendations.

Australia has national consensus guidelines for adults with FN [24] but only local (hospital-based or state-based) guidelines for children, with no national guidelines. Our study, with a mixed population of children with solid and haematological tumours from cancer centres around the country (which strengthens its generalisability), lends support for formation of national paediatric FN guidelines. Data from our study suggest these should not routinely recommend empiric aminoglycoside therapy for children with FN, in keeping with recent international guidelines [7].

As this study is an analysis of real-life rather than randomised antibiotic prescribing in a prospective cohort of children with FN, unrecognised confounding factors may have affected patient outcomes, and this must be acknowledged as a limitation. Although it is clear from practice and our study that aminoglycosides may be given to “sicker” patients, when we adjusted for clinically relevant factors by which clinicians judge this, those given aminoglycosides still had worse outcomes. Factors we adjusted for include those specified in guidelines to determine recommendation for aminoglycoside use in these children. Apart from nephrotoxity, potential mechanisms for harm include release of bacterial endotoxin, hypersensitivity reactions, drug interactions, complications related to intravenous access, selection for resistant organisms and microbiome disturbance, among others. However we do not have data from this study to explain potential mechanisms of harm observed with early aminoglycoside prescribing found here, though complications of aminoglycoside therapy in this population have been described elsewhere.

As recommended doses of aminoglycosides vary in international guidelines it is possible that adverse outcomes may not have been seen had lower doses been routinely administered. Also, the precise durations of aminoglycosides prescribed here are unknown, and this would have been a useful measure by which to stratify our analysis. Our study was conducted in tertiary paediatric hospitals within Australia and some results may not be generalisable to children and adolescents treated in primarily adult hospitals or in countries where resistance patterns are substantially different. For example, results may not be generalisable to settings with higher rates of resistance to beta-lactams given for febrile neutropenia. Prescribers were free to choose therapy, which varied between and within sites, and thus this study type does not replace a need for prospective well-conducted trials to guide future antimicrobial practice and supportive care in cancer. Nonetheless, the size and richness of data here do allow for hypothesis generation and opportunities to better understand real-world paediatric cancer care.

## Conclusions

We found substantial variation in antibiotic prescribing for FN among the PICNICC cohort, including with regard to empiric aminoglycoside recommendations and practice. In this cohort, early aminoglycoside use appeared harmful while lack of aminoglycoside use was not associated with worse outcome, even in those who met guideline criteria for empiric aminoglycosides. Further efforts should be directed towards standardising recommendations for paediatric FN nationally, establishing the role for empiric aminoglycosides in this group, if any, and researching barriers and facilitators of efforts to reduce unnecessary variation in antimicrobial therapy for children with cancer.

## Supporting information

S1 Table Aminoglycoside receipt in those who met and who did not meet criteria for use in the 1st 12 hours by state. (PDF)

S1 Fig Outcome-free survival to 30 days by treatment group. Log-rank test for equality of survivor functions P = 0.0006.
(PDF)

S2 Fig Balance plot before and after propensity matching. (PDF)
