# Supplementary material for: Aminoglycoside use in paediatric febrile neutropenia – Outcomes from a nationwide prospective cohort study
Source: PLoS One. 2020 Sep 16;15(9):e0238787. doi: 10.1371/journal.pone.0238787 (PMC7494114; doi:10.1371/journal.pone.0238787)
Supplement: S1 Table — (PDF) [file pone.0238787.s001.pdf]

S1 Table. Aminoglycoside receipt in those who met and who did not meet criteria for use in the 1st 12 hours by state

**Qualified for aminoglycoside[a]**<br><br>**Total qualified = 400 (46.6%)**						
**State[b]**	**VIC**<br><br>**N (%)**	**QLD**<br><br>**N (%)**	**NSW**<br><br>**N (%)**	**SA**<br><br>**N (%)**	**WA**<br><br>**N (%)**	**Total**<br><br>**N (%)**
**Received aminoglycoside**	115 (53.5)	6 (37.5)	94 (58)	1 (25)	0 (0)	216 (54)
**Did not receive aminoglycoside**	100 (46.5)	10 (62.5)	68 (42)	3 (75)	3 (100)	184 (46)
**Did not qualify for aminoglycoside[a]**<br><br>**Total did not qualify = 458 (53.4%)**						
**State[b]**	**VIC**<br><br>**N (%)**	**QLD**<br><br>**N (%)**	**NSW**<br><br>**N (%)**	**SA**<br><br>**N (%)**	**WA**<br><br>**N (%)**	**Total**<br><br>**N (%)**
**Received aminoglycoside**	26 (12.3)	11 (7)	0 (0)	2 (3.2)	0 (0)	39 (9)
**Did not receive aminoglycoside**	186 (87.7)	146 (93)	0 (0)	61 (96.8)	26 (100)	419 (91)
**Total**	**427**	**173**	**162**	**67**	**29**	**858**

[a]Qualified=met local criteria for aminoglycoside

[b]State: VIC=Victoria, QLD=Queensland, NSW=New South Wales, SA=South Australia, WA=Western Australia
