# Supplementary material for: Aminoglycoside use in paediatric febrile neutropenia – Outcomes from a nationwide prospective cohort study
Source: PLoS One. 2020 Sep 16;15(9):e0238787. doi: 10.1371/journal.pone.0238787 (PMC7494114; doi:10.1371/journal.pone.0238787)
Supplement: S1 Fig — Log-rank test for equality of survivor functions P = 0.0006. (PDF) [file pone.0238787.s002.pdf]

S1 Fig. Outcome-free survival to 30 days by treatment group

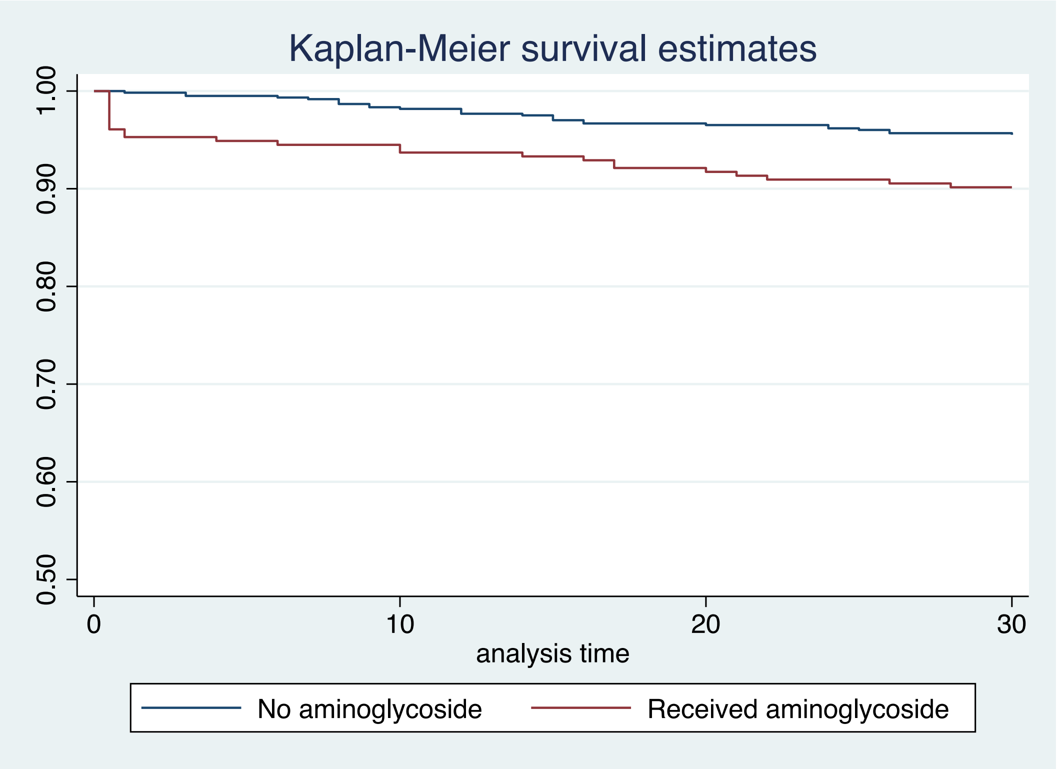

Log-rank test for equality of survivor functions P=0.0006
