# Supplementary figures and images for: Aminoglycoside use in paediatric febrile neutropenia – Outcomes from a nationwide prospective cohort study
Source: PLoS One. 2020 Sep 16;15(9):e0238787. doi: 10.1371/journal.pone.0238787 (PMC7494114; doi:10.1371/journal.pone.0238787)

**S2 Fig. Balance plot before and after propensity matching**

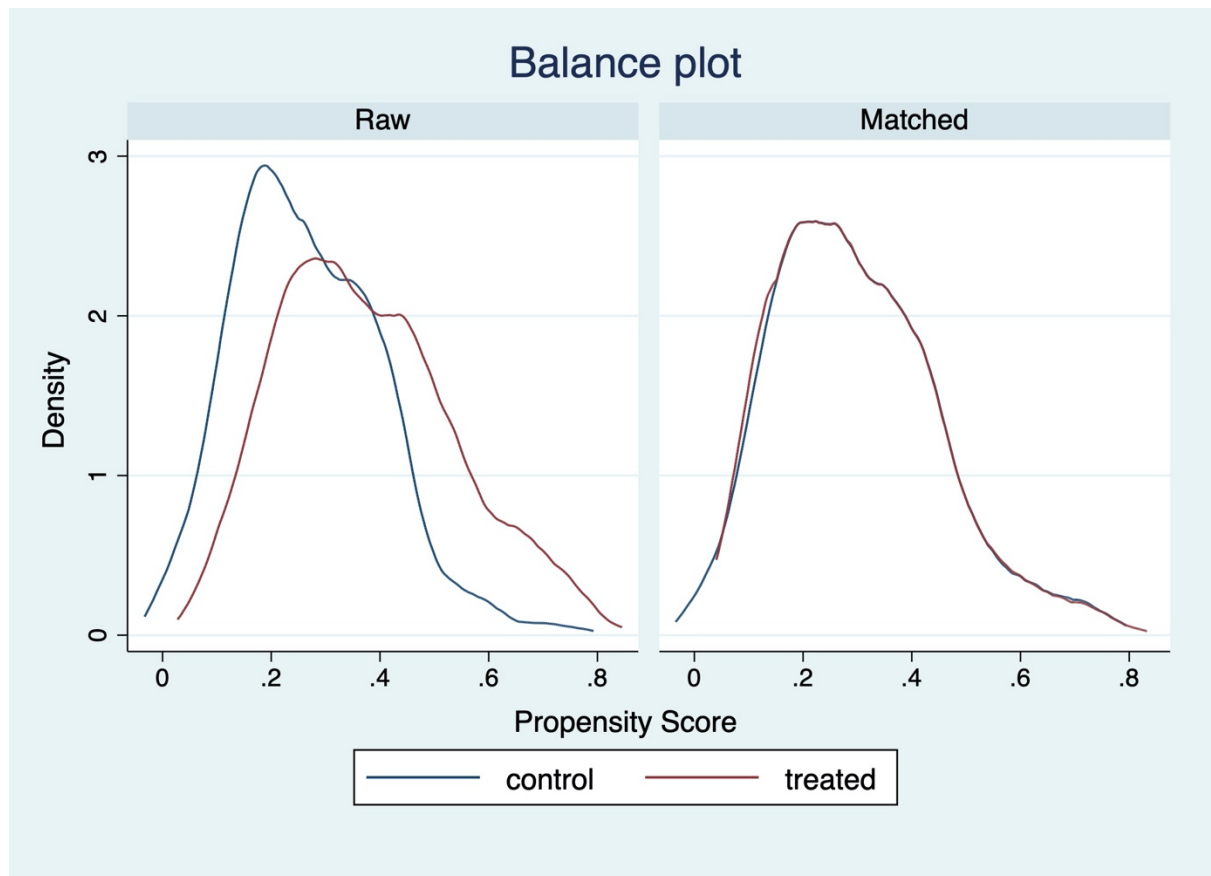

Supplement: S2 Fig — (PDF) [file pone.0238787.s003.pdf]
